# The Effect of Mother Goat Presence during Rearing on Kids’ Response to Isolation and to an Arena Test

**DOI:** 10.3390/ani11020575

**Published:** 2021-02-23

**Authors:** Louise Winblad von Walter, Björn Forkman, Madeleine Högberg, Eva Hydbring-Sandberg

**Affiliations:** 1 Department of Anatomy, Physiology and Biochemistry, Faculty of Veterinary Medicine and Animal Science, Swedish University of Agricultural Sciences, SE-750 07 Uppsala, Sweden; madeleine.hogberg@slu.se (M.H.); eva.sandberg@slu.se (E.H.-S.); 2 Department of Veterinary and Animal Science, Section of Animal Welfare and Disease Prevention, University of Copenhagen, 1870 Frederiksberg C, Denmark; bjf@sund.ku.dk

**Keywords:** animal welfare, behavior, caprine, cortisol, heart rate, fear, fear test, rearing system, ruminants, startle effect

## Abstract

**Simple Summary:**

The early permanent separation of mother and young in dairy production is the subject of much consumer concern. The aim of this study was to examine how early permanent separation, separation during the day only, or full-time access to their mother over two months affected goat kids, as measured by heart rate, saliva cortisol, and behavior during social isolation and exposure to a novel test situation. Our study shows that goat kids separated early permanent, separated daytime, or kept full-time with their mothers showed different responses to a challenge. However, it is difficult to say which of the treatments resulted in most fear and stress for goat kids during the tests. It seems that kids permanently separated from their mothers adapted to live in a group with other goat kids by the age of two months, while those separated daytime from their mothers demonstrated the strongest stress response. The reaction of goat kids kept full-time with mother fell between those of the other two treatments. In conclusion, the evaluation of stress responses is complicated, and our study pinpoints the importance of combining physiological with behavioral measurements.

**Abstract:**

The aim of the study was to examine how early permanent separation, separation during the day only, or full-time access to the mother goat affected goat kids during social isolation with a sudden sound of a dog bark at two weeks and two months, and a novel arena test with a novel object at two months. Kids permanently separated reduced their vocalization earlier and had a higher heart rate before and after dog bark during isolation at two weeks, no effect was found on the daytime separated kids. Daytime separated kids bleated more at two weeks and decreased heart rate after dog barking at two months. Daytime separated kids showed the strongest fear reaction in the arena test, no effect was found on the permanently separated kids. Kids separated early vocalized more before novel object and showed more explorative behavior afterwards. Our study shows different responses in goat kids separated early permanent, daytime separated, or kept full-time with mother, which demonstrates the importance of if and how the mother is present, and the impact of using a wide variety of physiological and behavioral measures when evaluating stress in animal welfare research.

## 1. Introduction

The common dairy industry practice of permanently separating mother from offspring, at an early age, has attracted increasing concern and criticism [1,2,3,4,5,6,7,8,9,10,11] and early permanent separation has been reported as stressful for both mother goats and their kids [12,13,14,15,16]. Maintaining the mother-offspring relationship is likely to have positive consequences for the welfare of the dam and goat kids [17,18]. The main argument put forward for early permanent separation is the increased amounts of milk collected from separated dairy goats [19,20] and cows [21,22]. However, in a recently published comprehensive review article on cow–calf interaction [23] prolonged contact with the suckling calf did not show a clear cut reduction in saleable milk. In addition, suckling most often had a positive effect on calf growth [23]. We have recently shown that goats, kept with their kids for sixteen hours per day, had higher milk fat, casein concentration, and curd yield compared with goats kept together with their kids for eight hours per day [24].

Goats show a large diversity in post-partum behavior [25] but are, like cattle, usually considered “hiders” early in life [25,26,27,28]. The time a goat kid spends hiding differs between populations and range from two days to approximately two weeks [29]. After the hiding phase, goat kids start to follow mother and are closely attached to her [28]. Still, except for the suckling bouts, goat kids synchronize their behavior with other kids rather than mother until two-three months of age, whereafter they start to synchronize with mothers behavior [15,30,31]. At one week of age, kids in some populations start to form social groups with other kids [29,30]. However, the formation of social groups depend on several factors, such as presence of similarly aged juveniles, predator pressure, herd density, the existence of a cohesive female herd, and is not seen in all populations [29].

With respect to behavioral development and animal welfare, Bungo et al. [32] suggested that weaning should not occur before six to seven weeks of age. Among feral goats, kids remain with their mothers from birth until eleven or twelve months of age [15]. Goats are highly social and vocal animals [33,34,35] and goats and kids establish an exclusive bond rapidly after birth [15,36,37,38]. Vocalization helps mother goats to locate their kid, and is also important in establishing the relationship between mother goats and their kids [35,36].

In arid parts of the world, offspring commonly have part-time access to their mother in dairy production [8]. A system of part-time suckling has been to some extent adopted by European farmers, which raises the need for a scientific evaluation of rearing systems featuring different degrees access to mothers for both cattle [8] and small ruminants. The effects of separation during the day with restricted contact between dam and goat kids and restricted suckling have been previously studied from the perspective of milk production and kid growth [17], and suckling has also been shown to improve milk quality in goats [24]. Restricted suckling is considered to be a management system advantageous for the welfare of dairy animals, since it allows for prolonged contact with the mother [7,24].

Isolation is a situation highly associated with fear and discomfort in goats [39,40,41] and arena tests are considered to be reliable fear tests for goats [40]. In this study, we wanted to identify how three different rearing treatments affect goat kids regarding fearfulness and welfare when separated. Therefore, we combined physiological and behavioral measures in goat kids during an isolation test, at two weeks and two months, and an arena test, featuring a suddenly appearing novel object, at two months. Thus, the aim of the study was to establish whether kids reared together with, in part with, or totally without their mother differed in their physiological and behavioral reactions to an aversive situation. We hypothesized that goat kids reared with their mothers for two months would be less fearful in their physiological and behavioral reactions and would show more explorative behaviors during aversive situations, when compared to early permanent separated kids. The reactions of daytime separated kids were expected to fall between reactions of the other two treatments.

## 2. Materials and Methods

### 2.1. Animals, Housing, and Management

Twelve pregnant goats (*Capra hircus*) were kept in an indoor pen (10 m × 7 m), with straw and wood shavings for bedding material, and tables and boxes provided as enrichment and hideouts. The goats were fed hay ad libitum and concentrates and carrots in conjunction with milking between 07:00 h and 08:00 h and 15:00 h and 16:00 h. Both kids and goats had free access to hay. The animals were fed in groups and no individual measurements were made. Water and mineral blocks were available ad libitum. The body weight gain was similar in all treatments and the results are included in another paper [42]. The study was carried out at the Swedish University of Agricultural Sciences in Uppsala. All animals were well accustomed to handling, and the care of the animals and the experimental design were both approved by the Animal Ethics Committee in Uppsala, Sweden (C 36/9). The animals also participated in a second study, and details concerning the animals are reported in [42].

### 2.2. Experimental Procedure

Before parturition, goat kids were randomly assigned to three future treatments: SEP (permanently separated from their mother after the colostrum period), DAY-SEP (daytime separated, in which goats and kids remained together, but separated between (7.30 h–15.00 h), and NON-SEP (no separation, in which goats and kids were kept together). In the case of twins or triplets, one kid was allocated to the same treatment as their mother (preferably female offspring), while siblings were allocated to SEP treatment. About one month separated the first parturition from the last. A total of 11 goats and 22 kids participated in the study (Table 1).

All kids suckled during the colostrum period of 4 days. Ten kids, nine males and one female, were thereafter separated from their mothers permanently (SEP). They were moved to another pen (4.5 m × 3 m and equipped with plastic boxes and tables) in the same animal room as the goat pen, where they were fed goat milk ad libitum from a self-feeder. The goats and kids in NON-SEP and DAY-SEP treatments were kept together in the home-pen. Goats usually are considered “hiders” two days to approximately two weeks in life [25,26,27,28] and thereafter start to follow their mother [28]. Goat kids synchronize their behavior with other kids rather than mother until two–three months of age [15,30,31]. Therefore, we wanted to study treatment effects during an early isolation test at two weeks, when the kids had been in their respective treatments for ten days, and during an isolation test and an arena test when the kids were two months old, and by this age should be more dependent on other kids and mother.

#### 2.2.1. Isolation Test at Two Weeks of Age

When the kids were two weeks old, an isolation test was performed. The isolation box was 2.4 m × 2.4 m, and the walls consisted of bar grids covered with plywood boards 1.1 m high. The kids were moved to the isolation box and isolated for 12 min in total. To study not only the effect of isolation but also the effect of isolation at a stressful event a dog bark was played from a computer after 10 min, and the goat kids were observed for two minutes further. During isolation, the same individual made direct observations, in the form of continuous recordings of vocalizations. After the isolation, the kids that were separated from their mothers (SEP) moved back to their home pen, and the kids that were daytimeseparated (DAY-SEP), and not separated (NON-SEP) were reunited with their mothers in a box (1.2 m × 1.5 m) adjacent to their home pen for ten minutes. Thereafter, the kids were moved to their home pen together with their mothers. During the isolation, heart rate was measured by telemetry.

#### 2.2.2. Isolation and Novel Arena Test at Age Two Months

At two months of age, an additional isolation test and an arena test were performed. The kids were randomly assigned to begin with either the isolation test, or the arena test. During the arena test, behavioral observations, saliva sampling, and heart rate measurements were performed. The test arena was 4.8 m × 4.8 m, and the walls consisted of bar grids covered with plywood boards 1.1 m high. The arena test was combined with another commonly used stressor, a novel object test [40]. A novel object, i.e., a plastic bag filled with cans, was attached to the ceiling with a rope. The kids were in the arena for twenty minutes. After ten minutes, the novel object was released, falling to the floor, and the kids were studied for an additional ten minutes. After the arena test, all kids were moved back to their home pens.

#### 2.2.3. Behavioral Observations

During isolation and the arena test, the same individual made direct observations in the form of continuous recordings of vocalizations. The rest of the behaviors exhibited during the arena test (Table 2) were recorded by video cameras mounted at each side of the novel arena (Panasonic, Osaka, Japan). The recordings were analyzed in Boris version 4.1.1 [43] by the same individual.

#### 2.2.4. Heart Rate

Throughout all the tests, heart rate was measured telemetrically using the Polar Sport tester for human beings (Polar Vantage NV^TM^, Polar Ltd., Bromma, Sweden). In order to maximize contact between the electrodes and skin, electrode gel (Blågel; Cefar Medical Products AB, Lund, Sweden) was applied to the belt of the heart rate monitor before placing it around the goats’ chests. To further increase contact, the belt was fastened with Vetrap (3M Animal Care Products, St. Paul, MN, USA). The heart rate was registered every fifth second. The data was transferred to a computer using the software Polar ProTrainer 5.

#### 2.2.5. Saliva Cortisol

Saliva samples were taken with Salivettes (Sarstedt, Germany) by the same individual (LW). Four saliva samples were taken from each kid during the isolation test. The first sample was taken in their home pen on the morning of the isolation test, the second sample was taken immediately after completion of the isolation test, and the third sample was taken ten minutes after reunion with mother or the kids group, respectively, and the fourth sample was taken one hour after reunion.

Three saliva samples were taken from each kid during the arena test. The first sample was taken in their home pen on the morning of the arena-test, the second sample was taken immediately after the test was finished, and the third sample was taken one hour after the test.

The Salivettes were centrifuged for 20 min at 5000× g, and the samples were stored at −20 °C until analysis. Analyses of saliva cortisol were performed using a commercial radio-immuno-assay kit (Coat-A-Count, radioimmunoassay, Diagnostic Product Corporation, Los Angeles, CA, USA) validated for goat saliva with a recovery of 93%. The intra-assay coefficient of variation was ≤10% between 0.8 and 110 nmol/L, inter-assay low 4.2 nmol/L (9.98%), medium 14.8 nmol/L (10.4%), high 30.1 nmol/L (1.2%), and the least detectable value was 0.8 nmol/L.

### 2.3. Statistics

Behavioral and hormonal data are presented as means ± S.E.M. Data were examined using the repeated measurement ANOVA (mixed procedure) of the Statistical Analysis System (SAS Institute Inc. Cary, NC, USA, 2003). The statistical model included the effect of sample, system, gender, and animal. For analysis of heart rate and vocalization data in the isolation test, the heart rate and vocalization values were divided in six periods (0–2 min, 2–4 min, 4–6 min, 6–8 min, 8–10 min, and 10–12 min after dog bark). For analysis of heart rate and behavioral data in the arena test, the test was divided in three periods: before novel object (1–10 min), novel object (minute 10–11), and after novel object (11–20 min). Differences between genders are only presented when overall significances were found. Pairwise comparisons within treatment were tested for significance using differences in least square means (the DIFF option). The level of significance was set at *p* ≤ 0.05.

## 3. Results

See Supplementary Materials for data on isolation and arena test. 

### 3.1. Isolation Test

#### 3.1.1. Heart Rate and Vocalization during Isolation—Comparisons between Treatments at Two Weeks

**Figure 1:**
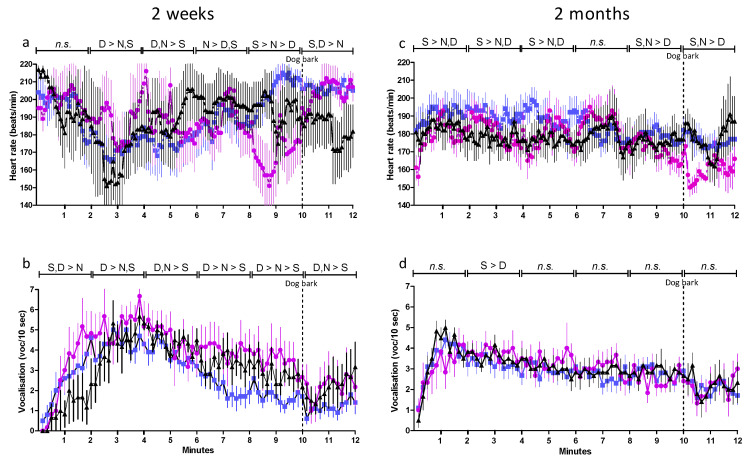
Heart rate and vocalization frequency at two weeks (**a**,**b**) and two months (**c**,**d**) (means± SEM) in SEP kids (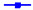) DAY-SEP kids (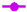) and NON-SEP kids (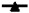) during 12-min isolation test with the sound of a dog bark at 10 min. SEP = (S) 10 kids separated from their mother, DAY-SEP = (D) 6 kids separated from their mother between 7.30 h–15.00 h, and NON-SEP = (N) 6 kids kept together with mothers. > indicates significant differences between treatments stated in the figure. The level of significance was set at *p* ≤ 0.05.

Both heart rate and vocalization showed large individual variation and fluctuated considerably during the test. There were no significant differences in heart rate during the first two minute period (Figure 1a), but SEP kids (*n* = 10) and DAY-SEP kids (*n* = 6) vocalized significantly more than NON-SEP kids (*n* = 6, *p* ≤ 0.05, Figure 1b). During the subsequent two-minute period (2–4), DAY-SEP kids had both higher heart rates and vocalization rates, compared with the other two treatments (Figure 1a,b). Between four and six minutes (4–6), both DAY-SEP and NON-SEP kids had higher heart rates and vocalization rates, compared with SEP kids (Figure 1a,b). Between six and eight minutes (6–8), NON-SEP kids had the highest heart rates, and DAY-SEP kids the highest vocalization rates (Figure 1a,b). Later in the test (minutes 8–12), SEP kids had the highest heart rates and lowest vocalization rates (Figure 1a,b). In contrast to the other treatments, the heart rate decreased after the dog bark in NON-SEP kids.

#### 3.1.2. Heart Rate and Vocalization during Isolation—Comparisons between Treatments at Two Months

SEP kids had higher heart rate than both DAY-SEP and NON-SEP kids during the first six minutes (Figure 1c). During the subsequent two minutes (6–8), there was no difference between treatments, but during the last four minutes both SEP and NON-SEP kids had higher heart rates than DAY-SEP kids (Figure 1c). The vocalization rate did not differ between treatments, except for a transient elevation in SEP kids compared with DAY-SEP kids during minutes 2–4 (Figure 1d).

#### 3.1.3. Heart Rate and Vocalization during Isolation—Comparisons between Ages

The mean overall heart rate was higher during isolation at two weeks, compared with at two months, for all treatments (Figure 1a,c). The mean vocalization rate was higher at two months than at two weeks for SEP kids (Figure 1b,d). For DAY-SEP kids, the mean vocalization rate was higher at two weeks than at two months, and for NON-SEP kids there were no significant differences between ages (Figure 1b,d). Both heart rate and vocalization fluctuated less at two months than at two weeks.

#### 3.1.4. Saliva Cortisol during Isolation—Comparisons between Treatments at Two Weeks, and Two Months

**Figure 2:**
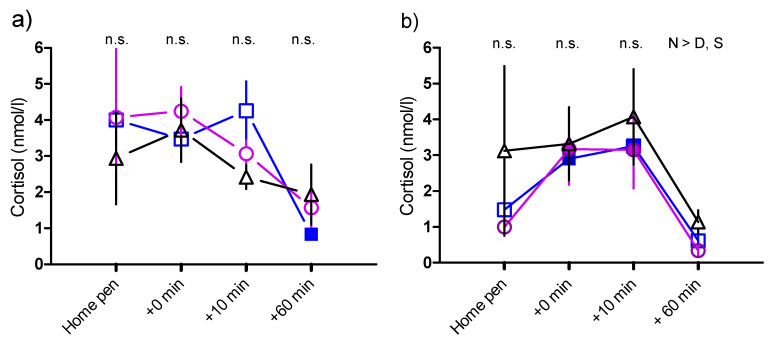
Saliva cortisol concentrations in nmol/L, (means ± SEM) in SEP kids (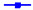) DAY-SEP kids (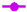) and NON-SEP kids (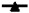) at 2 weeks (**a**) and 2 months (**b**) of age in home pen before isolation (Home pen), immediately after isolation, but before reunion (+0 min), 10 min after reunion with mother respectively kids group (+10 min), and 60 min after reunion (+60 min). SEP = (S) 10 kids separated from their mother, DAY-SEP = (D) 6 kids separated from their mother between 7.30 h and 15.00 h, and NON-SEP = (N) 6 kids kept together with mothers. > indicates significant differences between treatments stated in the figure. Filled symbols indicate that values are significantly different from the first sample. The level of significance was set at *p* ≤ 0.05.

At two weeks, the saliva cortisol concentration decreased in the last sample (60 min after reunion) in SEP goat kids compared to the first sample, but there were no differences between treatments (Figure 2a). At two months, the saliva cortisol concentration increased after isolation test and 10 min after reunion in SEP and DAY-SEP kids. In the last sample, taken in home pen one hour after reunion with the mother, or with the group of other kid goats, respectively, the cortisol concentration was higher in NON-SEP kids compared to SEP and DAY-SEP kids (Figure 2b).

### 3.2. Novel Arena Test

#### 3.2.1. Heart Rate during the Arena Test—Comparisons between Treatments

**Figure 3:**
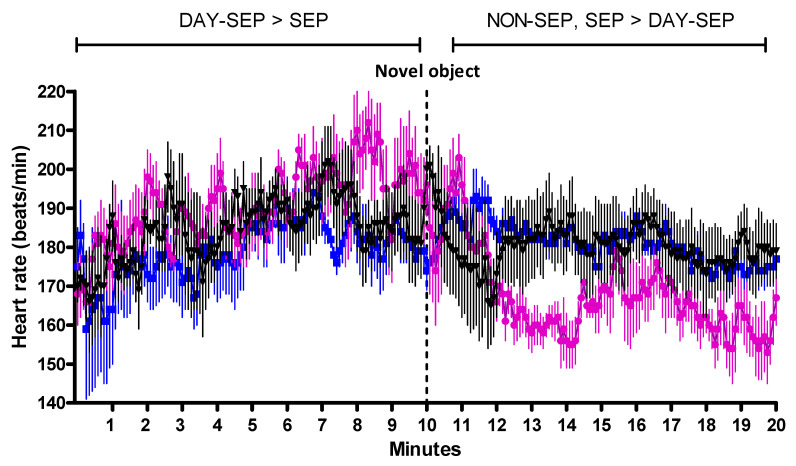
Mean heart rate (beats/min) in SEP kids (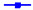) DAY-SEP kids (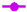) and NON-SEP kids (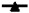) during an arena test. SEP = 10 kids separated from their mother, DAY-SEP = 6 kids separated from their mother between 7.30 h and 15.00 h, and NON-SEP = 6 kids kept together with mothers. The arena test lasted for 20 min, with a novel object appearing after 10 min. For purposes of comparison, the arena test was divided into two periods; before novel object (PRE = minute 1–10) and after novel object (POST = minute 11–20). > indicates significant differences between treatments stated in the figure. The level of significance was set at *p* ≤ 0.05.

During the first ten minutes before introduction of the novel object (PRE), the mean heart rate was higher for DAY-SEP kids than SEP kids, but fell below both the other treatments during the period after introduction of the novel object (POST) (Figure 3).

#### 3.2.2. Behavior during the Arena Test—Comparisons between Treatments

Before introduction of the novel object, SEP kids vocalized more than DAY-SEP kids, while DAY-SEP kids showed more locomotive behavior, entered more squares, jumped more times, and spent more time by the wall than SEP kids (Table 3). After introduction of the novel object, DAY-SEP kids spent more time by the wall than both SEP and NON-SEP kids, and SEP kids explored more than DAY-SEP kids (Table 3). The reaction of NON-SEP kids fell in between that of the two other treatments throughout the test.

#### 3.2.3. Heart Rate and Behavior—Reactions to Novel Object

DAY-SEP and NON-SEP kids had higher mean heart rates before introduction of the novel object than after. On the contrary, SEP kids had elevated mean heart rates after introduction of the novel object, compared with before (Figure 3).

Kids in all treatments were more active (vocalizing, locomotive behavior, jumping, entering more squares per minute, and exploring) before the novel object than after, but a greater proportion of observations found them by the wall, after introduction of the novel object, than before (Table 3). Locomotion was higher in SEP kids than DAY-SEP kids (Table 3). There was an overall gender effect in latency to sniff object, this taking longer for females than males (433 ± 86 s and 149 ± 83 s, respectively; *p* ≤ 0.05).

#### 3.2.4. Saliva Cortisol during the Arena Test—Comparisons between and within Treatments

Saliva cortisol concentrations did not differ between treatments, but was elevated in SEP kids after arena test compared to home pen (*p* ≤ 0.02) (Table 4).

## 4. Discussion

Our study clearly shows that goat kids that are early permanent separated, separated daytime, or kept full time with their mothers during their first two months of life show differing responses to a challenge. However, it is difficult to say in which of the treatments the kids were most fearful and stressed during the tests. Kids separated early permanent (SEP kids) deviated most from the other treatments in the isolation test at two weeks, by reducing their vocalization earlier and having a higher heart rate before and after the sound of a dog bark, and at two months, by having a higher heart rate throughout the test. On the other hand, DAY-SEP kids bleated comparatively more at two weeks, and showed a clear decrease in heart rate after the sound of a dog barking at two months. In addition, DAY-SEP-kids showed the strongest fear reaction in the arena test, performed at two months of age, showing increased escape behavior before “startle”, and by a clear drop in heart rate and greater passivity after the introduction of a novel object. In opposite, SEP kids vocalized more before the novel object but showed more explorative behavior after the novel object, even though this difference was not significant compared to NON-SEP kids.

Acoustic signals communicate that animals are “in need” of something, and vocalization is used to call for herd members [44], or to facilitate group contraction in goats [45], and thus has been used as a measure of acute distress in goat kids [46,47]. Hence, increased vocalization in goats during social isolation can be interpreted as a sign of fear or distress, and an adaptive attempt to communicate with flock mates. In line with other studies, where the initial response to isolation of goats was enhanced activity and increased vocalization [39,41,48], the vocalization rate during isolation in our study rapidly increased to peak level at both ages, and thereafter slowly decreased.

At two weeks, the vocalization rate after dog bark in DAY-SEP and NON-SEP kids increased, while the SEP kids continued vocalising at a lower level. The sound of a dog bark has been shown to cause alertness in isolated goats [49] and exposure to a dog caused increased vocalization and elevated blood pressure and plasma cortisol in lactating goats [50]. Hence, the dog bark probably initiated an enhanced fear response in the arena test. The lack of vocalization response to dog bark, and the overall lower vocalization rate in SEP kids, at two weeks, when compared to the other treatments, indicate that the kids housed with their mother, either part time or full time, used bleating as a means to reinstate contact with their mothers, while SEP kids may not have developed a similarly strong bond to their flock-mates at this young age. At two months, the vocalization rate was similar in all treatments, but the heart rate in SEP kids was elevated compared to the other treatments during a major part of the isolation test. The elevated heart rate may be explained by an enhanced stress reaction compared to the other treatments. It is possible that the lack of maternal care affected SEP kids capacity to cope when challenged. Goat kids develop a strong bond to their mothers [15,30,36,37,38] and mother goats and their kids show mutual recognition already during the hiding phase [34] and maternal care have a direct and strong influence on offspring survival in goats [51]. Hence, it is probable that the goat mother represents safety for the goat kids in challenging situations. For young lambs, Napolitano et al. [52] mean that the mother is the most relevant social model. Even though goat kids differ from lambs that are followers, mother’s care is most likely highly relevant for social development in goat kids. However, the elevated heart rate in SEP kids may also arise from a high level of locomotion in attempts to reinstate contact, compared to the other treatments, which in that case indicate that they had developed a strong bond to their flock mates by two months of age. According to Lickliter [26], goat kids in start to form subgroups with other kids approximately seven days after birth, and are closely associated to other kids already by two weeks of age. Even though this is not true for all populations [29], goat kids are probably motivated to form social bonds to other kids from seven days of age [28] and the only possible social contact for the permanently separated kids in our study was the other kids.

Social isolation is a known stressor in goats and induces such strong behavioral responses as increased vocalization and locomotion [39,41,48]. Therefore, vocalization [12], locomotor activity, and escape attempts [47] are used as indicators of stress during social isolation in goat kids. However, the interpretation of behaviors during isolation and the arena test is not self-evident. Active behaviors, like locomotion and vocalizations, may reflect a high motivation to reinstate social contact and a low level of fear, but might also be interpreted as indicative of fear [41,53,54]. In cattle, it has been demonstrated that inactive behaviors during the arena test (such as immobility or latency) seem to be correlated to fear-related reactions [53,54]. In our study, goat kids in all treatments fell silent, became passive and spent more time by the wall after novel object in the arena test. We suggest that an expected intense fear reaction to the novel object, caused a passive avoidance reaction in the form of behavioral inhibition. Upon threats, animals activate different defensive modes [55] and the behavioral response to a fearful situation varies with both active and passive strategies [40], where passive strategies are expressed by immobility or movement inhibition [53]. Freezing, a form of behavioral inhibition, is accompanied by an activation of the parasympathetic nervous system which decreases heart rate [56], whereas active fear responses are usually associated with an activation of the sympathetic nervous system and increased heart rate [55]. At two months isolation, and in the arena test, the heart rate in DAY-SEP kids decreased after the dog bark and introduction of the novel object, respectively, which indicates a parasympathetic activation. Interestingly, before introduction of the novel object, the DAY-SEP kids were more physically active, including jumps against the wall, and had a higher heart rate compared to SEP kids. Jumps against the wall are considered measures of escape attempts, and an indication of fear during the arena test by gregarious animals [40]. Hence, the elevated heart rate in DAY-SEP kids may be caused by a combination of physical activity and fear. In a study by Wagner et al. [57], dairy calves reared with their mothers showed more escape attempts during isolation than calves reared with other calves. This is to some extent in line with the present study, where DAY-SEP kids showed more escape attempts than SEP kids. However, this was not true for NON-SEP kids.

The over-all reaction to the isolation test was generally stronger at two weeks of age than two months, except for the SEP kids, who vocalized more at two months than two weeks. According to Siebert et al. [41], goats do not habituate to social isolation, which implies that the generally higher heart rate at two weeks, compared to two months, was probably not a habituation effect. Adult goats have lower heart rate than goat kids [58], thus, the lower heart rate at two months may be an age effect.

Cortisol concentration and heart rate are commonly used as physiological indicators of stress in goats [39,46,50,59,60,61,62,63]. Overall, concentrations of saliva cortisol were low in all samples, with large individual differences. One single difference between treatments was found in the last sample, at the two-weeks isolation test, where NON-SEP kids had a slightly greater concentration than the other treatments. This is difficult to explain, and probably not of biological relevance, due to the low levels and large individual differences. The intention of measuring saliva cortisol was to use a non-invasive method in the young goats, since restraint during blood sampling can cause an elevated cortisol level [28]. Contrary to Kannan et al. [61], but in line with Carbonaro et al. [39], we did not find any significant cortisol responses to the arena test. However, in SEP and DAY-SEP kids, we found a significant cortisol response to isolation at two months. We expected a cortisol response to isolation and the arena test in all treatments, and the reason why cortisol concentrations did not change at two weeks, or in NON-SEP kids at two months, may be the high pre-test value. The high value may be a handling effect, although the kids were trained.

The only overall gender difference we found was in the arena test, where the latency to sniff object was longer in females than in males. Chojnacki et al. [47] found that female goat kids exposed to prenatal stress, were more fearful than male goat kids during a social and separation test, but Andersen et al. [28], found no differences between genders in behavioral response to social isolation. Since our intention in the herd was to keep the female goats for recruitment, we chose to keep the female kids with their mothers for this study. This is in accordance with most farmers, who likely choose to keep a female kid with the goat, since they are intended to remain in the herd. In our study, however, this led to a skewed distribution of gender between treatments.

In this study, we investigated a few short-term effects of isolation in the rearing systems with or without mother in goats. However, there is a lack of knowledge concerning the long-term effects on behavior and physical performance of rearing with or without the mother in goats. Studies of cattle suggest that prolonged dam-contact may enhance social abilities in heifers [7]. However, Zipp and Knierim [64] found no long-term effects on physical development in heifers reared with whole-day contact, half-day contact, or no contact with their mothers, despite increased growth in nursed calves. Nevertheless, dam-reared heifers lay more than the non-contact heifers when introduced to the herd, which the authors suggested indicates better adaptation to the situation. Such studies would be of great interest to goat dairy producers, and their consumers.

## 5. Conclusions

It seems that kids permanently separated from their mothers had developed a bond to their flock mates and thereby adapted to live in a group with other goat kids by the age of two months, while those separated daytime demonstrated the strongest stress response at this age. Contrary to our hypothesis, the reaction of the non-separated kids fell between the other two treatments. Our study shows that goat kids separated early permanent, separated daytime, or kept full-time with their mothers showed different responses to a challenge. This demonstrates the importance of if and how the mother is present, as well as the impact of using a wide variety of physiological and behavioral measures when evaluating stress in animal welfare research. For future research and development, it is desirable to focus on housing and management systems, with special consideration of the positive effects of keeping mother and offspring together.

## Figures and Tables

**Table 1:** Participating goats and kids in the different treatments.

Goat	Lactation	Kids, nr and Gender	Treatment
1	3	1, ♂	DAY-SEP
		2, ♂	SEP
2	1	3, ♀	NON-SEP
		4, ♂	SEP
3	1	5, ♂	DAY-SEP
		6, ♂	SEP
		7, ♂	SEP
4	3	8, ♀	NON-SEP
		9 ♂	SEP
5	2	10, ♀	DAY-SEP
6 ^1^	2	11, ♂	SEP
		12, ♂	SEP
7	2	13, ♀	NON-SEP
		14, ♀	SEP
8	3	15, ♀	NON-SEP
		16, ♂	SEP
9	3	17, ♂	DAY-SEP
		18, ♂	SEP
10	2	19, ♀	NON-SEP
11 ^2^	3	20, ♀	DAY-SEP
		21, ♀	DAY-SEP
12	2	22, ♂	NON-SEP

^1^ euthanized nine days after parturition due to udder problems, ^2^ Stayed together with two kids.

**Table 2:** Definitions of observed behaviors in kids, during an isolation test at two weeks and two months of age, and during an arena test at two months of age.

Behavior	Description	Continuous Recording
Vocalization	The kid making sound, bleating	Counts per 10 s
Number of squares entered	New square was counted when entering an aligned square with one forefoot	Counts per 10 s
Jumping/rearing	Jumping against wall with all four feet, or rearing against wall with forefeet	Counts per 10 s
Startle reaction	Number of squares entered, and time from novel object until the kids were standing still more than 5 s	Counts of squares entered and seconds
Latency to sniff novel object	Latency between novel object on the floor and sniffing with muzzle less than 5 cm from novel object	Seconds
		Instantaneous sampling
Location in arena	Wall: Located in one of the squares close to the wall	Proportion of observations
Standing	Standing on all four feet	Proportion of observations
Locomotion	Moving except jumping (walking, trotting, running or climbing bars) Standing still and walking/running are mutually exclusive	Proportion of observations
Exploring	Sniffing with the muzzle less than 5 cm from wall or floor	Proportion of observations

**Table 3:** Mean values ± SE for behavior during an arena test. SEP = 10 kids separated from their mother, DAY-SEP = 6 kids separated from their mother between 7.30 h and 15.00 h, and NON-SEP = 6 kids kept together with mothers. The arena test lasted for 20 min, with a novel object appearing after 10 min. For purposes of comparison, the arena test was divided into two periods; before novel object (PRE = minute 1–10) and after novel object (POST = minute 11–20). Different letters indicate differences between treatments within periods and within treatments between periods.

Treatment	SEP		DAY-SEP		NON-SEP			
Period	PRE	POST	PRE	POST	PRE	POST	*p*-Values ^1^	*p*-Values ^2^
Vocalizations No./10 s	2.49 ± 0.07 ^a^	0.80 ± 0.04 ^c^	2.10 ± 0.09 ^b^	0.65 ± 0.06 ^c^	2.40 ± 0.07 ^ab^	0.41 ± 0.04 ^c^	PRE 0.03 POST n.s.	SEP 0.0001 D-S 0.0001 N-S 0.0001
Locomotion (proportion of observations)	0.32 ± 0.01 ^a^	0.23 ± 0.01 ^c^	0.40 ± 0.02 ^b^	0.10 ± 0.01 ^c^	0.35 ± 0.02 ^ab^	0.15 ± 0.01 ^c^	PRE 0.034 POST n.s.	SEP 0.0001 D-S 0.0001 N-S 0.0001
Squares entered No./minute	5.50 ± 0.37 ^a^	2.90 ± 0.3 ^c^	7.90 ± 0.52 ^b^	1.30 ± 0.35 ^c^	6.50 ± 0.50 ^ab^	2.10 ± 0.35 ^c^	PRE 0.048 POST n.s.	SEP 0.0001 D-S 0.0001 N-S 0.0001
Jumps No./minute	0.62 ± 0.10 ^a^	0.10 ± 0.03 ^c^	1.70 ± 0.24 ^b^	0.02 ± 0.02 ^c^	1.00 ± 0.20 ^ab^	0.06 ± 0.03 ^c^	PRE 0.0006 POST n.s.	SEP 0.0001 D-S 0.0001 N-S 0.0001
Located by the Wall (proportion of observations)	0.48 ± 0.02 ^a^	0.55 ± 0.02 ^c^	0.60 ± 0.02 ^b^	0.82 ± 0.02 ^d^	0.48 ± 0.02 ^ab^	0.63 ± 0.02 ^c^	PRE 0.02 POST SEP vs D-S 0.002 POST D-S vs N-S 0.03	SEP 0.002 D-S 0.0001 N-S 0.0002
Exploring (proportion of observations)	0.34 ± 0.02 ^a^	0.27 ± 0.01 ^b^	0.32 ± 0.02 ^a^	0.13 ± 0.01 ^c^	0.37 ± 0.02 ^a^	0.23 ± 0.02 ^bc^	PRE n.s. POST 0.02	SEP 0.002 D-S 0.0001 N-S 0.0001

^1^ Differences between treatments as indicated by differing superscript letters, ^2^ Differences between PRE and POST periods within treatments as indicated by differing superscript letters.

**Table 4:** Mean values ± SE for saliva cortisol concentrations (nmol/L) during the arena test. SEP = 10 kids separated from their mother, DAY-SEP = 6 kids separated from their mother between 7.30 h and 15.00 h, and NON-SEP = 6 kids kept together with mothers. The arena test lasted for 20 min, with a novel object appearing after 10 min. Different letters indicate differences between treatments within periods and within treatments between periods; *p* ≤ 0.05.

Treatment	SEP	DAY-SEP	NON-SEP
Home pen	1.0 ± 0.2 ^a^	1.2 ± 0.2 ^a^	0.9 ± 0.3 ^a^
After arena test	1.9 ± 0.5 ^b^	2.5 ± 0.7 ^ab^	1.9 ± 0.7 ^ab^
+60 min	0.8 ± 0.1 ^a^	1.0 ± 0.3 ^a^	0.7 ± 0.3 ^a^

## Data Availability

The data presented in this study are available as supplementary material.

## Supplementary Materials

The following are available online at https://www.mdpi.com/2076-2615/11/2/575/s1, Table S1: Isolation heart rate, Table S2: Isolation vocalisation, Table S3: Isolation cortisol, Table S4: Arena test heart rate, Table S5: Arena test vocalisation, Table S6: Arena test cortisol, Table S7: Arena test behaviour.
