# Supplementary figures and images for: The Effect of Mother Goat Presence during Rearing on Kids’ Response to Isolation and to an Arena Test
Source: Animals (Basel). 2021 Feb 23;11(2):575. doi: 10.3390/ani11020575 (PMC7926452; doi:10.3390/ani11020575)

2 weeks

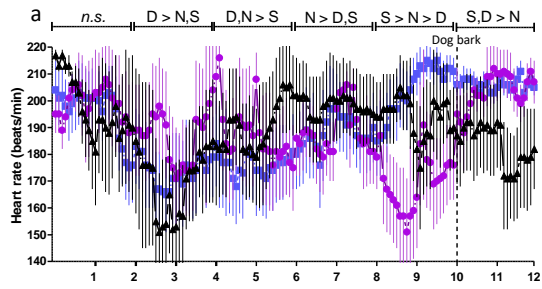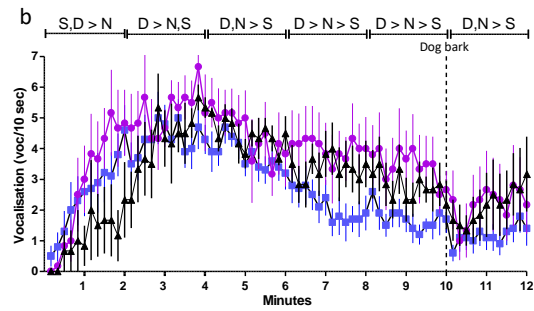

2 months

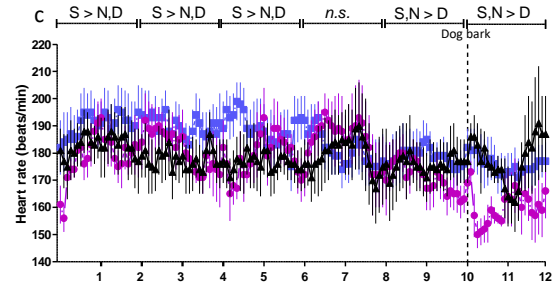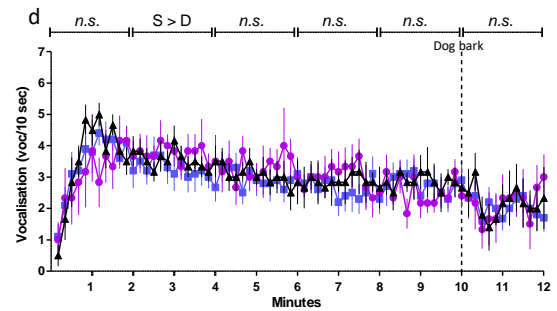

Supplement: Supplementary file 1 [file animals-11-00575-s001.zip › Figure 1.pdf]

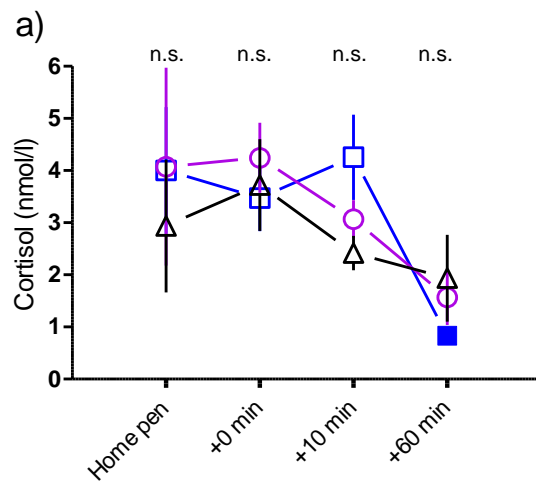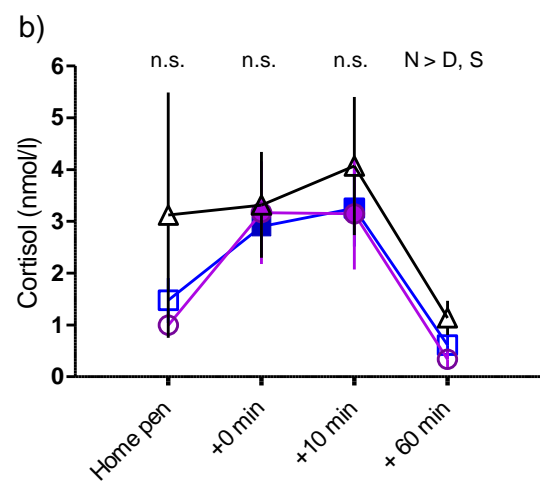

Supplement: Supplementary file 1 [file animals-11-00575-s001.zip › Figure 2.pdf]

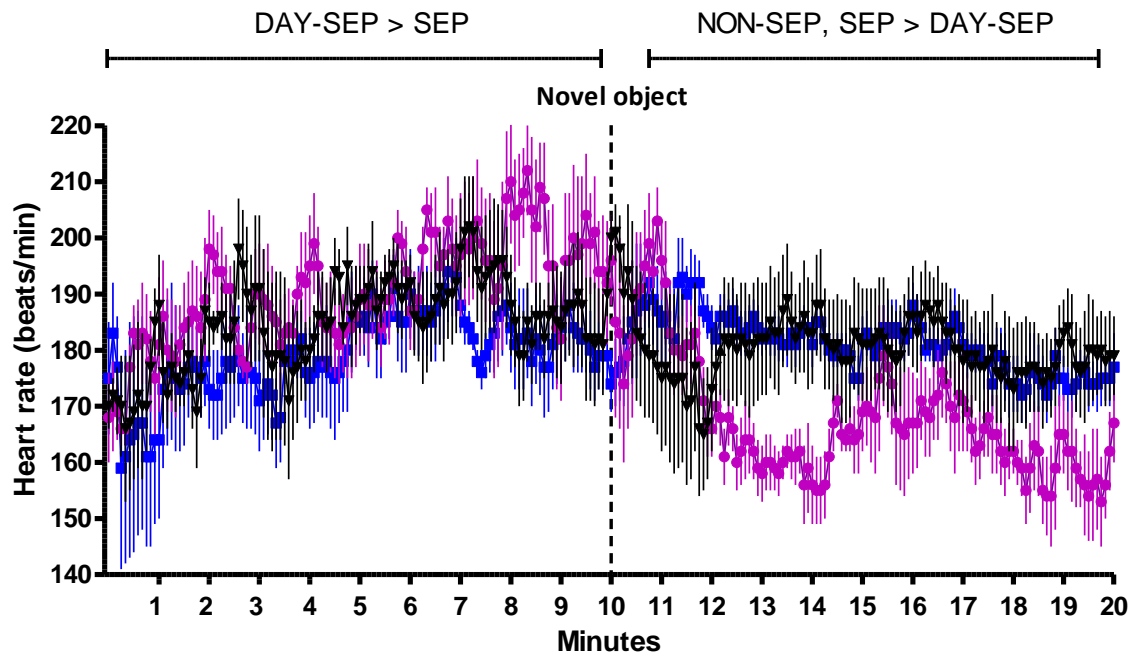

Supplement: Supplementary file 1 [file animals-11-00575-s001.zip › Figure 3.pdf]
